# Clinical and Translational Considerations for Understanding Depression and Anxiety in Patients with Inflammatory Bowel Disease

**DOI:** 10.1155/2021/6689443

**Published:** 2021-03-05

**Authors:** Ashley M. Sidebottom, Tina G. Rodriguez, Jordan E. Karpin, David T. Rubin

**Affiliations:** ^1^Department of Medicine, University of Chicago, Chicago, IL, USA; ^2^University of Chicago Inflammatory Bowel Disease Center, Chicago, IL, USA

## Abstract

Depression and anxiety are comorbidities of inflammatory bowel disease (IBD). Though previous studies have proposed a relationship between anxiety, depression, and IBD, causality and directionality are largely unknown. Current and future research in these areas is aimed at exploring the biological underpinnings of this relationship, specifically pertaining to small molecule metabolism, such as tryptophan. Tryptophan is acquired through the diet and is the precursor to several vital bioactive metabolites including the hormone melatonin, the neurotransmitter serotonin, and vitamin B3. In this review, we discuss previous findings relating mental health comorbidities with IBD and underline ongoing research of tryptophan catabolite analysis.

## 1. Introduction

Depression and anxiety are observed at high rates in patients with chronic diseases, including inflammatory bowel disease (IBD). These psychiatric comorbidities impact patients' quality of life (QoL), their IBD treatment compliance, and morbidity [1–3]. Though previous work has shown a relationship between depression and anxiety and IBD, directionality and causality remain unknown.

Metabolites from the gastrointestinal (GI) tract impact and regulate systemic processes throughout the body and include many subclasses such as short chain fatty acids (SCFAs), lipids, secondary bile acids, and tryptophan catabolites. Tryptophan is acquired through the diet and is converted by intestinal microbes and the host via three main pathways: the indole pathway, the kynurenine pathway, and serotonin synthesis. Serotonin (5-hydroxytryptamine (5-HT)), a key neurotransmitter in the manifestation of depression and anxiety, is produced primarily in the digestive tract by epithelial cells and, to a lesser extent, in the brain following conversion. Although 5-HT cannot cross the blood brain barrier (BBB), precursors of 5-HT (i.e., L-kynurenine) are able to cross the BBB, and their concentrations have previously been reported to be altered in IBD patients. In this systematic review, we summarize available research on the relationship between IBD, mental health comorbidities, and tryptophan metabolism in the gut and peripheral tissues.

## 2. Methods

PubMed and Google searches were performed. Only original studies pertaining to the prevalence of depression and anxiety in an IBD cohort, the impact of these comorbidities on disease course, hospitalizations, and health-related quality of life (HRQoL), the importance of evaluating mental health in IBD, the effect of psychotropic medication on gut function, and the relationship between the microbiome and the manifestation of depression and anxiety were included.

## 3. Main Text

### 3.1. Clinical Considerations

Depression and anxiety are mental health disorders that affect patients with chronic diseases at a higher rate than the general age-matched population [4]. This trend holds true for IBD patients whose lifetime depression and anxiety rates are as high as 40% and 30%, respectively [5]. Among these patient populations, researchers found that women were more likely to have depression and anxiety; men were more likely to have undiagnosed depression and anxiety and that disease activity was associated with comorbid depression and anxiety [5, 6].

Recent studies by Blackwell and colleagues reported that patients experiencing gastrointestinal (GI) symptoms and have been diagnosed with depression were at increased risk for future UC or CD development compared to age-matched controls. Additionally, they report UC or CD patients had increased depression in the years prior to GI diagnosis [7]. In a complementary study by Karpin et al., the Computerized Adaptive Testing for Mental Health (CAT-MH), an adaptive testing technology, was utilized to measure and evaluate real-time depression and anxiety status that was compared against demographic and clinical variables. They determined patients who had clinically active disease were at a greater relative risk of having depression and anxiety compared to patients in remission [8]. Together, these studies suggest mental health status is closely linked to GI health status, and identification of key regulators of this relationship is essential for treatment development.

The impact of depression and anxiety on patients with IBD is far reaching, affecting their hospitalizations, health care costs, QoL, and disease course. The prevalence of anxiety and depression is often diagnosed with the use of various methods (i.e., HADS, SCID, and ICD-9) and in different settings (Table 1). In a sampling frame including more than 100,000 discharges, a comorbid diagnosis of depression was significantly associated with $30,000 more in total charges on average and an increased length of stay (LOS) of about 6 days [9]. Moreover, patients' disease course, QoL, and mental health status are all intimately related. Numerous studies describe lower QoL ratings and higher depression and anxiety scores when patients were in active disease state, an association between symptoms of depression and clinical recurrence of IBD, and a negative impact on their QoL [10–12]. This decreased QoL in patients with IBD may result from anxiety and fear associated with eating. Patients show altered eating habits due to the immense amount of information available regarding the diet's impact on IBD and the belief that certain foods can trigger disease flares. Evidence suggests a bidirectional relationship between diet, mental health, and disease severity and points to a need for further exploration of how these parameters interact with the gut microbiome [13]. The directionality and causality of mental health disorders and IBD are unknown, but this review is aimed at highlighting the current work that has been performed exploring this relationship.

Despite the aforementioned heightened prevalence of depression and anxiety in patients with IBD, these mental health comorbidities remain largely underdiagnosed and untreated [14]. Evertsz et al. used the Hospital Anxiety and Depression Scale (HADS) to screen patients for anxiety and depression, finding that although 42% of patients with IBD screened positive for depression and or anxiety, the majority of these patients had not previously used psychiatric medications or counseling services [14]. Utilization of mental health services can be predicted by other factors such as income, but it is important to note that only approximately 21.3% of IBD patients with a mental health issue currently seek mental health support [15]. Regular mental health screening of patients with IBD and appropriate management of patients with mental health comorbidities could greatly improve QoL. Such measures could include creating a network of local mental health professionals to whom to refer patients and implementation of IBD specific therapists or psychiatrists into clinic settings.

### 3.2. Pharmacological Considerations

Approximately one-third of patients with IBD have been prescribed antidepressants to ameliorate the effects of mental health comorbidities such as anxiety and depression [6]. In addition to targeting neural pathways in the brain, antidepressants such as serotonin reuptake inhibitors (SSRIs), tricyclic antidepressants (TCAs), and serotonin and norepinephrine inhibitors (SNRIs) have gastrointestinal functions that range from undesired side effects (i.e., constipation, diarrhea) to beneficial outcomes (i.e., increased gastric motility and decreased diarrhea). These compounds are reported to impact intestinal events through the regulation of neurotransmitters and neurotransmitter receptors, which are also involved in gut motility [16, 17]. The potential benefits of antidepressants in IBD was reported by Frolkis et al., where they describe patients with preexisting depression as more likely to be diagnosed with IBD and that antidepressant treatments proved protective against the development of Crohn's disease (CD) and ulcerative colitis (UC) [18]. Such findings identified the potential benefits of antidepressant use on disease course. Additionally, current evidence supports further benefits of SSRI drugs, including enhanced colonic phasic contractility and compliance, suppression of colonic tonic response to a meal, and shortened orocecal transit time [19, 20].

The large subset of patients with IBD receiving antidepressants highlights the importance of understanding the multifaceted relationship between psychiatric medications and gastrointestinal disorders such as IBD. Research exploring potential benefits of antidepressants on the course of disease and HRQoL has been inconclusive. For instance, though SSRI, fluoxetine, has been shown to ameliorate inflammation in a mouse model of colitis, this effect was not observed in a pilot clinical trial in humans with IBD [21, 22].

### 3.3. Translational Considerations

The central nervous system (CNS) and the intestinal microbiota influence and respond to each other through the endocrine and immune systems. Dysbiotic gut populations are often found in chronic IBD patients, such as CD and UC, and are associated with higher prevalence of mental disorders including anxiety and depression [6]. Despite the crucial role of microbes in disease onset, progression, and remission events, elucidation and treatment of principal host-microbe mechanisms acting on the hypothalamic-pituitary-axis (HPA, gut-brain-axis) remains a challenge.

Many factors influence brain disorder presentation such as neurotransmitter localization and regulation [23], circulating cytokine levels (IL-7, IL-10) [24], genetic polymorphisms (i.e., SERT, rs25531) [25], and the composition of the intestinal microbial community. Intestinal microbes can affect depression-like behaviors through several mechanisms including the alteration of immune response, metabolite conversion, and serum levels of bioactive compounds. In UC and CD patients with dysbiotic communities, studies report increased fecal diversity for patients diagnosed with depression [26] and decreased diversity for patients diagnosed with anxiety [27]. Specifically, in patients diagnosed with depression, a decrease in *Bifidobacteriaceae* [28], *Lachnospiraceae* [29], *Lactobacillaceae* [28], *Ruminococcaceae* [26], and *Veillonellaceae* [30] was observed with an increase in *Bacteroidales* [29], *Enterobacteriaceae* [26], and *Rikenellaceae* [26]. Additionally, patients diagnosed with anxiety have decreased abundance of *Firmicutes*, *Veillonellaceae*, *Prevotellaceae*, and *Tenericutes* [27]. Importantly, although these findings are meaningful, the significance and function of a microbe can vary between subjects and within the same subject over time making association by 16S rRNA-based assignment to phenotypic or disease outcomes informative but ultimately limited in mechanistic understanding.

Work by Valles-Colomer and coworkers recently analyzed the Flemish Gut Flora Project (*n* = 1,054) and determined how QoL was impacted by communities from IBD and depression patients [30]. Through metagenomic analysis, they were able to identify distinct, variable mechanisms in patients with IBD such as the production of a dopamine downstream metabolite, 3,4-dihydroxyphenylacetic acid (DOPAC), which was associated with a higher QoL. Additionally, they determined decreased microbially derived alpha-aminobutyric acid was associated with increased depression. Findings such as these are crucial in the development of targeted therapies. Additional studies focused on the incorporation of traditional methods with metagenomics, metatranscriptomics, and/or metabolomics are needed to identify community-wide functions within a host that are accessible for the development of therapeutic interventions.

**Figure 1:**
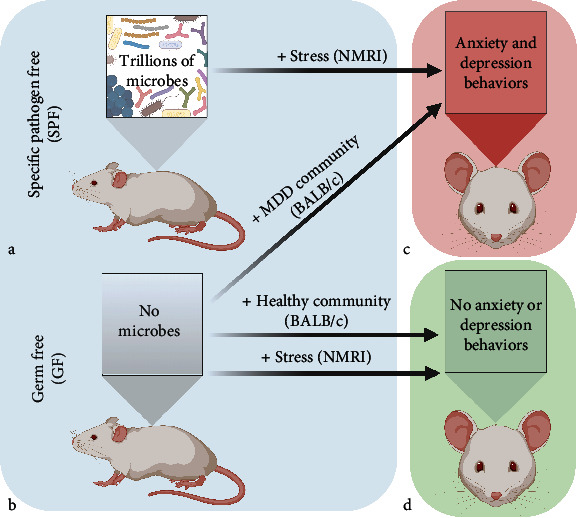
Anxiety and depression SPF and GF mouse models. (a) Specific pathogen free (SPF) mice are exposed to microbes and develop mature populations containing trillions of microbes throughout their body (e.g., skin, genital, and intestinal tract). SPF colonized mice have mature immune systems and are often used as the wild-type microbial community background in murine studies. (b) Germ free (GF) mice are bred without exposure to any microbes and are often used to study microbial community or specific population member (monocolonization) impact on host health. SPF and GF mice can be studied on varied genetic backgrounds (e.g., BALB/c and NMRI). (c, d) When exposed to stress (+Stress), SPF mice develop anxiety and depression behaviors while GF mice do not display these behaviors under stress or in the presence of a healthy community (+Healthy Community). GF mice do display anxiety and depression when colonized with a human microbial community from patients with major depressive disorder (+MDD Community) (figure created with http://BioRender.com).

The study of microbial community impact on a host is often completed *in vivo* with murine models bred without exposure to microbes (germ-free (GF)) which are often compared to animals with a “normal” murine microbiome (i.e., specific pathogen free (SPF)) (Figures 1(a) and 1(b)). With this approach, studies have reported decreased anxiety and depression-like behaviors in GF compared to conventionalized SPF when the animals are exposed to stress (Figures 1(c) and 1(d)) [31, 32]. Additionally, anxiety/depression behaviors can be introduced to a host through gut microbial community transplantation as demonstrated by Luo and coworkers (Figures 1(b) and 1(c)) [33]. In their study, antianxiety and antidepression GF BALB/c mice were conventionalized with a microbial community derived from major depressive disorder (MDD) patients [34]. Following colonization of these microbes, the animals displayed anxiety and depression and alterations in the glucocorticoid pathway indicating a microbial community can distinctly impact host brain disorders in a host that was previously not susceptible to disorder development. Additional studies identified that decreased fecal diversity of *Firmicutes* and increased *Gammaproteobacteria* was associated with IBD and depression [35]. It will be necessary moving forward to determine the microbial community components that influence and respond to host factors (i.e., genetic susceptibility, immune system status, and treatment history).

**Figure 2:**
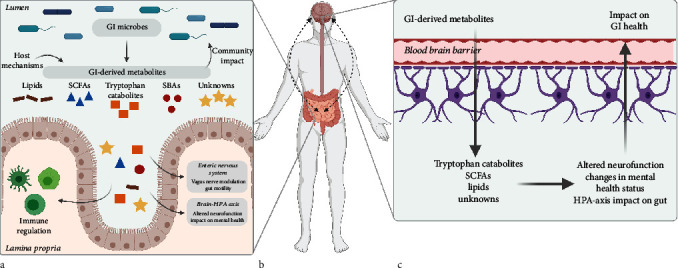
Overview of gastrointestinal-derived metabolites and their systemic impact. (a) GI microbes and host mechanisms drive production of metabolites that modulate processes such as immune function. Additionally, these molecules are transported systemically and impact the enteric nervous system and the brain-HPA axis. (b, c) Metabolites from the GI tract are transported to the brain, and those able to pass the BBB may impact neurofunction such as neurotransmitter receptor expression and mental health status. Changes in brain activity are then transported back to the GI tract which has been shown to impact functions such as gut motility and nutrient uptake. SCFAs: short chain fatty acids; SBAs: secondary bile acids; GI: gastrointestinal; HPA: hypothalamic-pituitary-axis (figure created with http://BioRender.com).

The intestinal environment affects systemic functions most notably through metabolites derived from the host, diet, and microbiome (Figure 2(a)). Systemically, many GI-derived metabolites can alter the HPA resulting in bidirectional impact on the gut from the brain, although the brain-based signaling pathways are not as well understood as those originating from the gut (Figure 2(b)) [6]. Within the intestinal tract, metabolites regulate mechanisms such as immune function, nutrient uptake, intestinal barrier function, and defense against invading microbes [36–38]. These compounds vary in physicochemical properties (i.e., size and hydrophobicity) and function in a wide range of mechanisms such as quorum sensing, nutrient metabolism, and host-microbe interactions and include major classes such as short-chain fatty acids (SCFAs), tryptophan catabolites, and secondary bile acids [35, 37, 39]. The tryptophan catabolites are often studied in brain disorder models because tryptophan is a precursor for the neurotransmitter, serotonin (5-hydroxytryptamine), and the hormone, melatonin. Tryptophan is derived from the host diet and is converted by intestinal microbes into several metabolites (i.e., kynurenine, serotonin, and additional indoles) which influences the local gut environment and are transported to the brain for further conversion into neurotransmitters (Figures 2(b) and 2(c)**)**. Studies show altered tryptophan metabolism is associated with increased inflammation in CD and UC patients [40]. Additionally, tryptophan catabolites are ligands for the aryl hydrocarbon receptor (AhR) pathway, a key regulator of the immune response in the gut, and they induce IL-22 and IL-10 production. The tryptophan catabolites impact the immune response and the microbial community in the intestine [41].

The function of tryptophan and downstream neurotransmitters, such as serotonin and melatonin, in anxiety and depression has been extensively studied, but the link between these disorders and IBD remains not well understood. Although distinct metabolite ratios such as kynurenine/tryptophan and serotonin/tryptophan have been reported to increase in IBD, it is unclear what role the microbiome plays in these levels and if these changes impact mental health [42]. Recent work by Singhal and colleagues reported the deletion of murine serotonin transporter (SERT^−/−^) which caused dysbiotic gut community formation in murine models and the induction of metabolic syndrome compared to wild-type controls. However, they did not assess the impact of this deletion on anxiety or depression [43].

### 3.4. Clinical Implications

Although the directionality of a proposed IBD and mental health disorders link via gut microbiome is not known, it is reasonable to presume that the alterations of the biome associated with inflammation may be directly contributing to the CNS and behavioral manifestations of depression and anxiety. Along these lines, it is possible that mental health disorders in some patients with IBD are not a result of the cognitive or social ramifications of having a chronic intestinal condition but rather are directly related to the inflammation itself. If true, the diagnosis and treatment of anxiety and depression in patients with IBD may be radically shifted to a focus on assessment of the gut microbiome, its neurotransmitter contributions, and the treatment of inflammation. Modern therapies for IBD that modify the gut immune system and achieve stable deep remission would be expected to also restore diversity of a healthy gut microbiome and in turn, modify the neurotransmitter components of depression and anxiety. Treatment of these mental health disorders in patients with IBD may end up being as much about bowel health as it is about TCAs and SSRIs. The bidirectional relationship between mental health and gut health is mediated by the gut-brain axis; just as gut inflammation may influence mental health, mental health may affect disease in the gut. The identification of key regulators along this axis will allow for more targeted treatment.

## 4. Conclusion

Further work is required to elucidate how the microbiome influences mental health disorders, and several factors should be considered, including early life perturbations, host genetic predispositions, and host-specific microbiome function and environment. It is clear gut microbes and their metabolic products play a critical role in mental health disorder presentation, and additional studies are needed to determine how microbes impact neurotransmitter synthesis/regulation/recognition, immune function, and if microbial communities from patients with IBD that do not display anxiety and depression behaviors can inform treatment or mental health diagnosis.

## Figures and Tables

**Table 1:** The prevalence of depression and anxiety in various IBD cohorts.

Reference	Depression and/or anxiety (%)	Diagnosis timeline	Assessment method	Diagnosis setting	Study type	Patient sample size
Lewis et al. [5]	Depression: 11.2% Anxiety: 16.9%	Current and lifetime	SCID	N/A	Cohort study	242
Byrne et al. [6]	Depression: 25.8% Anxiety: 21.2% D and/or A: 30.3%	Current	PHQ-9 and GAD-7 or diagnosis resulting from psychiatric interview	Tertiary care academic hospital	Retrospective chart review	327
Blackwell et al. [7]	Depression: UC, 3.7%, CD, 3.7%	5 years prior to IBD diagnosis	UK read codes	Primary care	Retrospective chart review	UC: 10,829 CD: 15,360
Stobaugh et al. [9]	Depression: 0.97%	Lifetime	ICD-9 codes	Hospital	Sampling frame from the NIS	100,687
Calixto et al. [10]	Depression (moderate): 55% anxiety (moderate): 41%	Current	HADS	Tertiary care center	Cross-sectional study	120
Mikocka-Walus et al. [11]	Depression: 20.2% Anxiety: 37.5%	Current	HADS	Mixed: hospitals and private practice	Prospective cohort study	2007
Ishak et al. [12]	Depression: 7.3% Anxiety: 18.2%	Current	PROMIS GHS, PROMIS-29, SF-12, WHODAS 2.0	Tertiary care center	Cross-sectional study	110
Bennebroek Evertsz et al. [14]	Depression: 6.3% Anxiety: 17.5%	Current	HADS and SF-12	Tertiary care center	Prospective cohort study	231
Knowles et al. [15]	Mental health issue (mild to severe): 51.8%	Current	K10	Online	Cross-sectional study, online	336

IBD: inflammatory bowel disease; D: depression; A: anxiety; PHQ: Patient Health Questionnaire; GAD: general anxiety disorder; UK: United Kingdom; CAT-MH: Computerized Adaptive Testing for Mental Health; ICD-9: International Classification of Diseases, Ninth Revision; HADS: Hospital Anxiety and Depression Scale; PROMIS GHS: Patient-Reported Outcomes Measurement Information System Global Health Survey; WHODAS: WHO Disability Assessment Schedule; SCID: Structured Clinical Interview for Axis I Disorders; NIS: Nationwide Inpatient Sample; GHS: Global Health Scale; SF: Short Form Health Survey; K10: Kessler Psychological Distress Scale.

## Data Availability

The data supporting this review are from previously reported studies and datasets, which have been cited in the text.
